# Variation in both host defense and prior herbivory can alter plant-vector-virus interactions

**DOI:** 10.1186/s12870-019-2178-z

**Published:** 2019-12-16

**Authors:** Xiaobin Shi, Evan L. Preisser, Baiming Liu, Huipeng Pan, Min Xiang, Wen Xie, Shaoli Wang, Qingjun Wu, Chuanyou Li, Yong Liu, Xuguo Zhou, Youjun Zhang

**Affiliations:** 1 Hunan Academy of Agricultural Sciences, Institute of Plant Protection, Changsha, 410000 China; 2 Institute of Vegetables and Flowers, Chinese Academy of Agricultural Sciences, Beijing, 100081 China; 3 Department of Biological Sciences, University of Rhode Island, Kingston, RI 02881 USA; 4 Hunan Horticultural Research Institute, Hunan Academy of Agricultural Sciences, Changsha, 410125 China; 5 Institute of Genetics and Developmental Biology, Chinese Academy of Sciences, Beijing, 100101 China; 6 Department of Entomology, University of Kentucky, Lexington, KY 40546 USA

**Keywords:** Virus-vector-host interaction, Plant defense, Jasmonic acid, Plant volatile, *Bemisia tabaci*

## Abstract

**Background:**

While virus-vector-host interactions have been a major focus of both basic and applied ecological research, little is known about how different levels of plant defense interact with prior herbivory to affect these relationships. We used genetically-modified strains of tomato (*Solanum lycopersicum*) varying in the jasmonic acid (JA) plant defense pathways to explore how plant defense and prior herbivory affects a plant virus (tomato yellow leaf curl virus*,* ‘TYLCV’), its vector (the whitefly *Bemisia tabaci* MED), and the host.

**Results:**

Virus-free MED preferred low-JA over high-JA plants and had lower fitness on high-JA plants. Viruliferous MED preferred low-JA plants but their survival was unaffected by JA levels. While virus-free MED did not lower plant JA levels, viruliferous MED decreased both JA levels and the expression of JA-related genes. Infestation by viruliferous MED reduced plant JA levels. In preference tests, neither virus-free nor viruliferous MED discriminated among JA-varying plants previously exposed to virus-free MED. However, both virus-free and viruliferous MED preferred low-JA plant genotypes when choosing between plants that had both been previously exposed to viruliferous MED. The enhanced preference for low-JA genotypes appears linked to the volatile compound neophytadiene, which was found only in whitefly-infested plants and at concentrations inversely related to plant JA levels.

**Conclusions:**

Our findings illustrate how plant defense can interact with prior herbivory to affect both a plant virus and its whitefly vector, and confirm the induction of neophytadiene by MED. The apparent attraction of MED to neophytadiene may prove useful in pest detection and management.

## Background

Approximately 80% of plant viruses, including many that damage or destroy economically-important crop species, are vectored by insects (1). Researchers are increasingly attuned to the importance of plant-virus-insect interactions, especially since plant pathogens have been shown to manipulate their vectors in ways that enhance their transmission and spread (2, 3). Despite substantial research, the complex nature of multi-species manipulations means that many important questions have yet to be addressed.

Plant responses to herbivore or pathogen attack are often mediated by the jasmonic acid (JA) metabolic pathway (4–6). For example, feeding by the spider mite *Tetranychus evansi* suppresses the release of plant volatiles and the JA signaling pathways in tomato (7). Similarly, bacteria in the oral secretions of Colorado potato beetle (*Leptinotarsa decemlineata*) larvae decrease production of JA and JA-responsive defenses in tomato (8). In virus-vector-host interactions, higher JA concentrations decrease feeding by whiteflies (*Bemisia* sp.) and reduce infections by whitefly-associated viruses (9, 10).

*Bemisia tabaci* Gennadius (Hemiptera: Aleyrodidae) is a phloem**-**feeding pest that includes at least 34 morphologically-indistinguishable but genetically distinct species (11). The *B. tabaci* Mediterranean (MED) is particularly invasive, and its recent colonization of China and other East Asian countries has caused severe economic losses. In addition to their feeding-related damage, whiteflies also vector plant viruses. MED is a particularly effective viral vector, and its invasion is often followed by disease outbreaks (12, 13). For example, tomato yellow leaf curl virus (TYLCV) causes crop loss worldwide (14), and its outbreaks in China have been linked to MED (reviewed in 13).

Scientists have made important progress in exploring plant*-*virus-*Bemisia* interactions (15). Zhang et al. (16) showed that tomato yellow leaf curl China virus (TYLCCNV) and its beta-satellite suppressed JA-based defense in tobacco against *B. tabaci* Middle East-Asia Minor 1 (MEAM1), and Luan et al. (17) found that TYLCCNV improved MEAM1 fitness by preventing whitefly-induced increases in terpenoid synthesis. While MEAM1 and MED occupy similar niches, they differ in a number of important ways (2, 18, 19) and research has shown that TYLCV infection of *Bemisia* host plants indirectly harms MEAM1 but benefits MED (20).

We report how JA-based plant defense, and its interaction with prior whitefly infestation, affect MED, TYLCV, and the MED-TYLCV-tomato interaction. We compared preference and performance of virus-free and viruliferous MED on tomato plants varying in their constitutive JA levels. We measured JA levels, and the expression of JA-related genes, in uninfested plants as well as those exposed to virus-free or viruliferous MED. We measured TYLCV titers in plants following exposures to viruliferous MED. We also analyzed how JA levels and whitefly infestation affect plant volatile emissions and MED preference. Our work illustrates how variation in both host defense and prior herbivory can individually and jointly alter the plant-vector-virus interaction. We also found a plant volatile compound that might prove useful as a whitefly attractant for use in new pest detection and management strategies.

## Results

### Experiment I: impact of JA-varying plant genotypes on virus-free and viruliferous MED

Feeding by viruliferous MED increased TYLCV levels in all three plant genotypes, while feeding of virus-free MED did not induce TYLCV levels. TYLCV loads in *spr2*, WT, and *35S* plants fed upon by viruliferous MED were 1.80 + 0.12 [SE], 1.33 + 0.18, and 0.87 + 0.26 O. D. 405, respectively. TYLCV loads in *spr2*, WT, and *35S* plants fed upon by virus-free MED as controls were 0.02 + 0.02, 0.04 + 0.03, and 0.03 + 0.01 O. D. 405, respectively.

Virus-free MED preferred low-JA *spr2* plants over high-JA *35S* plants. The *spr2* plants attracted 69 + 3.5 [SE]% of virus-free MED given the choice between them and *35S* (*p* < 0.001). Viruliferous MED exhibited a marginally-significant (*p* = 0.089) preference for low-JA *spr2* plants over high-JA *35S* plants: *spr2* plants attracted 63 + 6.7% of viruliferous MED given the choice between the low-JA genotype and *35S*. Neither virus-free nor viruliferous MED exhibited a preference between WT plants and either *spr2* or *35S* plants (*p* > 0.50 for all comparisons).

**Fig. 1:**
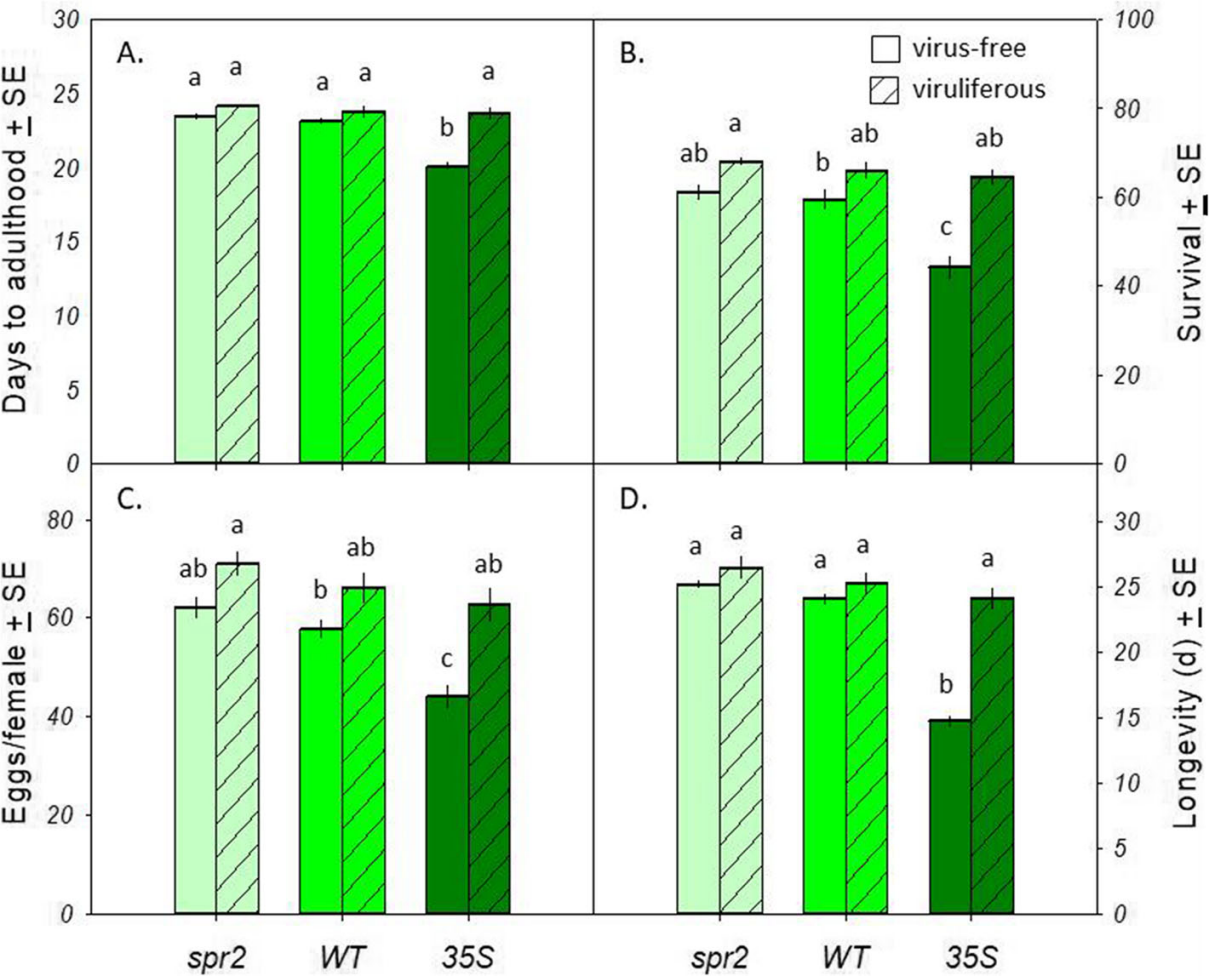
Impact of JA levels on virus-free and viruliferous MED. **A** Developmental time (days to adulthood). **B** Survival rate (percentage of population surviving to adulthood). **C** Fecundity (eggs per female). **D** Longevity (days as adult). *spr2*: tomato plants that underexpress JA; WT: wild-type tomato plants; *35S*: tomato plants that overexpress JA. Each treatment was replicated 30 times (30 virus-free and 30 viruliferous MED replicates) per genotype. Open bars: virus-free MED; striped bars: viruliferous MED. Among each group, bars with different lowercase letters are significantly different (*P* < 0.05)

High JA levels decreased the growth, survival, and fecundity of virus-free MED, but did not affect viruliferous MED (Fig. 1). Virus-free and viruliferous MED performed similarly (in terms of development time, survival rate, fecundity, and longevity) when feeding on the *spr2* and normal WT cultivars. On the *35S* cultivar, however, the fecundity, survival to adulthood, and adult longevity of virus-free MED was 30, 31, and 39% lower, respectively, than that of viruliferous MED (Fig. 1; Table 1A).

**Table 1:** Results of statistical analyses

A.	Plant genotype			MED status			Genotype*MED		
Response variable	*df*	*F*	*p*	*df*	*F*	*p*	*df*	*F*	*p*
Days to adulthood	2, 174	13.52	**< 0.001**	1, 174	45.13	**< 0.001**	2, 174	6.87	**< 0.001**
Survival to adulthood (%)	2, 174	15.26	**< 0.001**	1, 174	49.13	**< 0.001**	2, 174	7.85	**< 0.001**
Eggs/female	2, 174	12.62	**< 0.001**	1, 174	30.82	**< 0.001**	2, 174	2.39	0.094
Adult longevity (days)	2, 174	50.78	**< 0.001**	1, 174	51.95	**< 0.001**	2, 174	24.13	**< 0.001**
B.	Plant genotype			MED status			Genotype*MED		
Response variable	*df*	*F*	*p*	*df*	*F*	*p*	***df***	***F***	***p***
*LOX* relative gene expression	2, 18	59.86	**< 0.001**	2, 18	20.11	**< 0.001**	4, 18	16.65	**< 0.001**
*OPR3* relative gene expression	2, 18	3.12	0.069	2, 18	46.98	**< 0.001**	4, 18	13.82	**< 0.001**
*PI II* relative gene expression	2, 18	8.92	**0.002**	2, 18	41.28	**< 0.001**	4, 18	3.33	**0.033**
*JAR1* relative gene expression	2, 18	1.49	0.252	2, 18	40.65	**< 0.001**	4, 18	2.89	0.052
Jasmonic acid, ng/g	2, 18	307.5	**< 0.001**	2, 18	103.80	**< 0.001**	4, 18	39.10	**< 0.001**
C.	Plant genotype			Time			Genotype*time		
Response variable	*df*	*F*	*p*	*df*	*F*	*p*	***df***	***F***	***p***
*TYLCV* load post inoculation	2, 6	42.55	**< 0.001**	3, 18	3.79	**0.029**	6, 18	2.93	**0.036**

**Footnote: “*df*” refers to “degree of freedom”; “*F*” refers to “*F*** **value”; “*p*” refers to “*p*** **value”**

### Experiment II: JA-related genes and JA levels in three plant genotypes exposed to viruliferous and non-viruliferous whiteflies

Feeding by viruliferous MED increased TYLCV levels in all three plant genotypes, while feeding of virus-free MED did not induce TYLCV levels. TYLCV loads in *spr2*, WT, and *35S* plants fed upon by viruliferous MED were 2.03 + 0.21 [SE], 2.00 + 0.06, and 1.13 + 0.15 O. D. 405, respectively. TYLCV loads in *spr2*, WT, and *35S* plants fed upon by virus-free MED (TYLCV levels measured as control values) were 0.02 + 0.01, 0.05 + 0.03, and 0.03 + 0.02 O. D. 405, respectively.

**Fig. 2:**
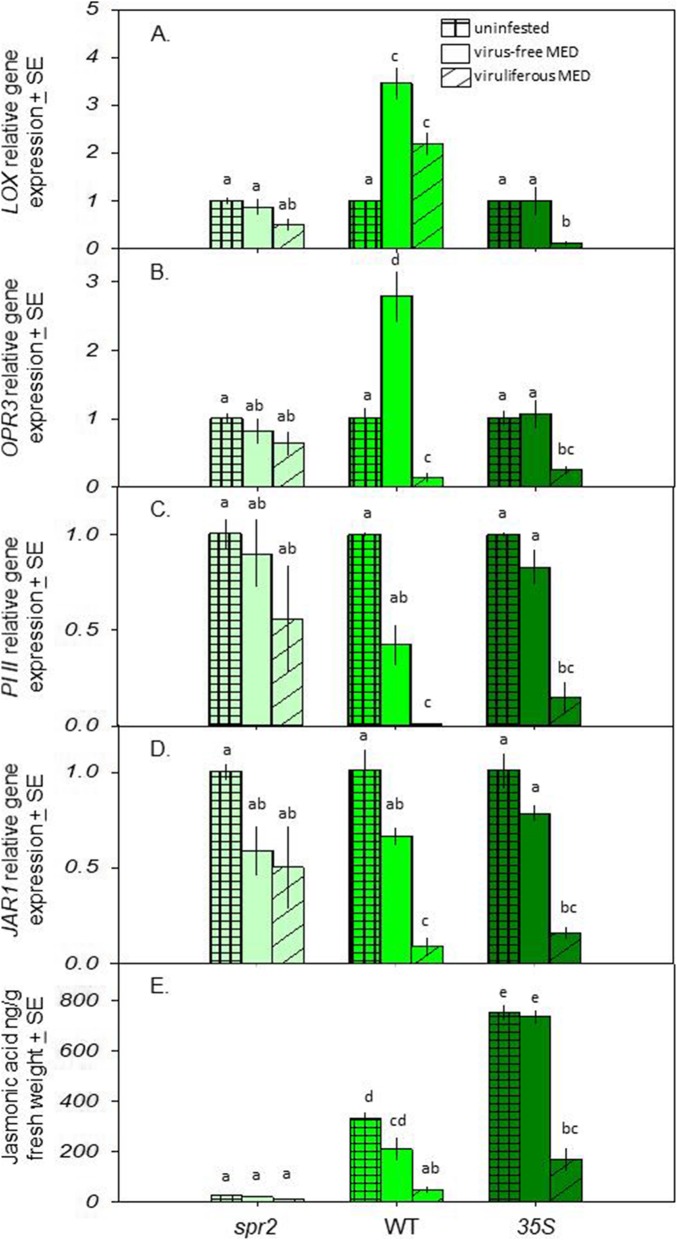
Impact of plant genotype and TYLCV infection on the expression of JA-related genes and JA levels in plant foliage*.* **A** *LOX* gene expression. **B** *OPR3* gene expression. **C** *PI II* gene expression. **D** *JAR1* gene expression. **E** JA concentration. Gene expression values are normalized to *ACT* and *UBI*. This protocol used 81 (three infestation treatments × three genotypes × nine replicates) plants. Plaid bars: non-infested control plants; open bars: plants infested with virus-free MED; striped bars: plants infested with viruliferous MED. Within each group, bars with different letters are significantly different (*P* < 0.05)

Virus-free MED feeding on WT plants increased expression of the ‘upstream’ (i.e., involved in JA biosynthesis) *LOX* and *OPR3* genes by 3.5x and 2.8x, respectively (Fig. 2A, B), but had no effect on the ‘downstream’ (i.e., induced by increased JA levels) *PI II* or *JAR1* genes (Fig. 2C, D; Table 1B). In contrast, viruliferous MED decreased expression of all four genes in *35S* plants by an average of 83% (*p* < 0.05 for all; Fig. 2A-D).

JA levels were lower in *spr2* versus *35S* plant genotypes, and in plants exposed to viruliferous MED (Fig. 2E). While virus-free MED did not reduce JA levels in any of the three plant genotypes, viruliferous MED reduced JA by 85% in WT plants and 77% in *35S* plants (*p* < 0.05 for both; Table 1C). Viruliferous MED did not reduce JA in *spr2* plants.

Virus-free and viruliferous MED did not affect the expression of JA-related genes in *spr2* plants. There was no effect of virus-free MED on the expression of JA-related genes in *35S* plants.

### Experiment III: impact of viruliferous MED on TYLCV titers and JA levels

**Fig. 3:**
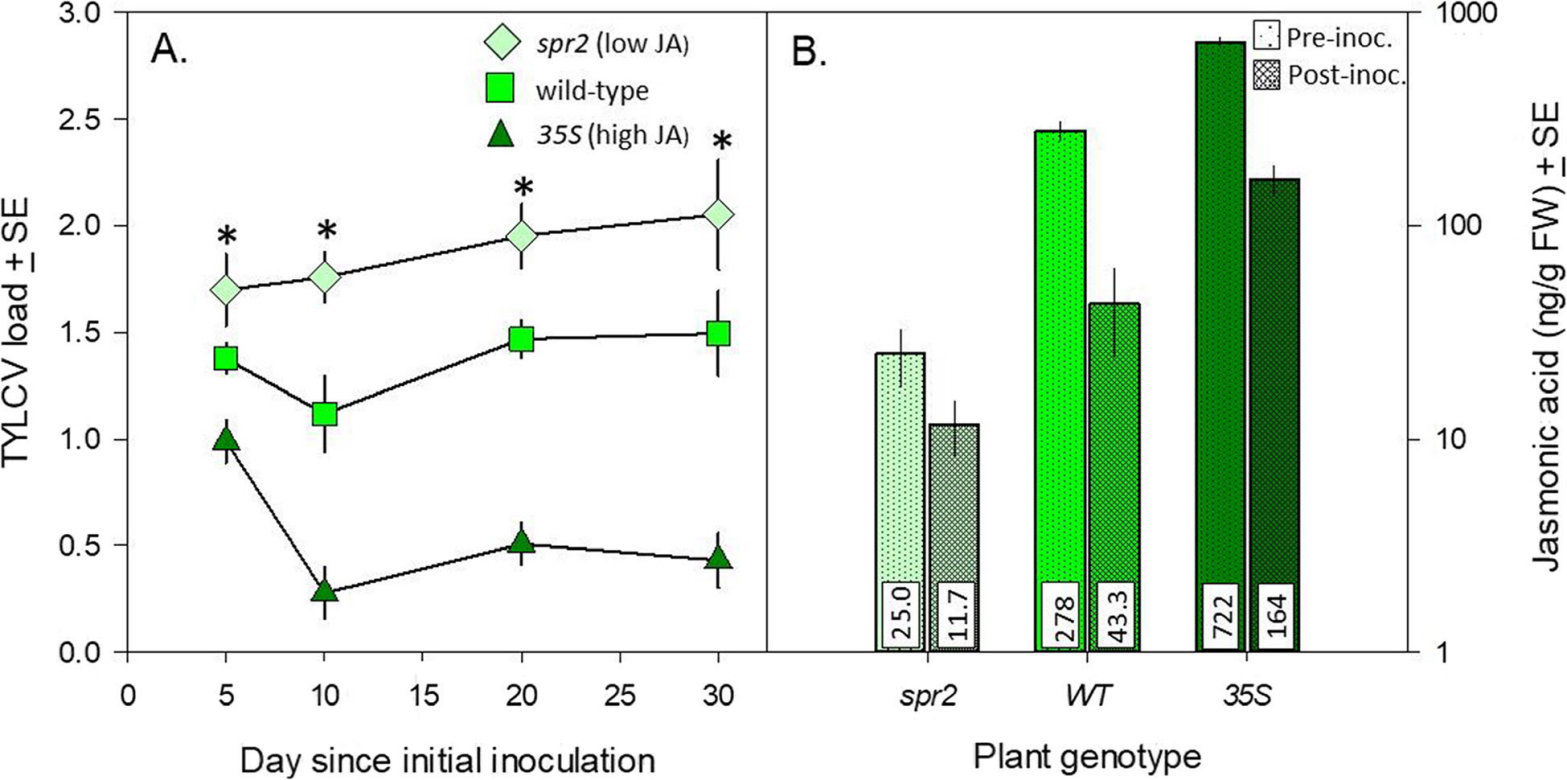
Impact of viruliferous MED on TYLCV titers and JA levels. **A** TYLCV titres in three plant genotype following inoculation with viruliferous MED; **B**. pre- and post-inoculation JA levels in each of the three plant genotypes (note log scale of Y-axis; mean JA values are presented in white inset bars at the base of each bar)*.* JA levels in plants were sampled one day before infestation and one day after infestation with viruliferous MED. The protocol for Fig. 3a used 36 (three genotypes × three replicates × four time points) plants. The protocol for Fig. 3b used 27 (three genotypes × three replicates × three time points) plants. Light green diamonds: *spr2*; bright green squares: WT; dark green triangles: *35S*. Asterisk indicates a significant difference in the three genotypes (*P* < 0.05)

Infestation with viruliferous MED produced the highest viral titers in the *spr2* plants, medium in the WT plants, and lowest in the *35S* plants (Fig. 3a; Table 1C). Viral titers also changed across time, reflecting a sharp drop in viral titers in the *35S* treatment following the first measurement. JA levels in all three plant genotypes decreased ~ 70% following infestation (Fig. 3B).

### Experiment IV: plant volatile emissions and MED preference as a function of genotype and prior MED infestation

Feeding by viruliferous MED increased TYLCV levels in all three plant genotypes, while feeding of virus-free MED did not induce TYLCV levels. TYLCV loads in *spr2*, WT, and *35S* plants fed upon by viruliferous MED were 2.33 + 0.09 [SE], 2.07 + 0.22, and 1.87 + 0.22 O. D. 405, respectively. TYLCV loads in *spr2*, WT, and *35S* plants fed upon by virus-free MED (serving as control plants) were 0.04 + 0.02, 0.03 + 0.02, and 0.05 + 0.03 O. D. 405, respectively.

**Fig. 4:**
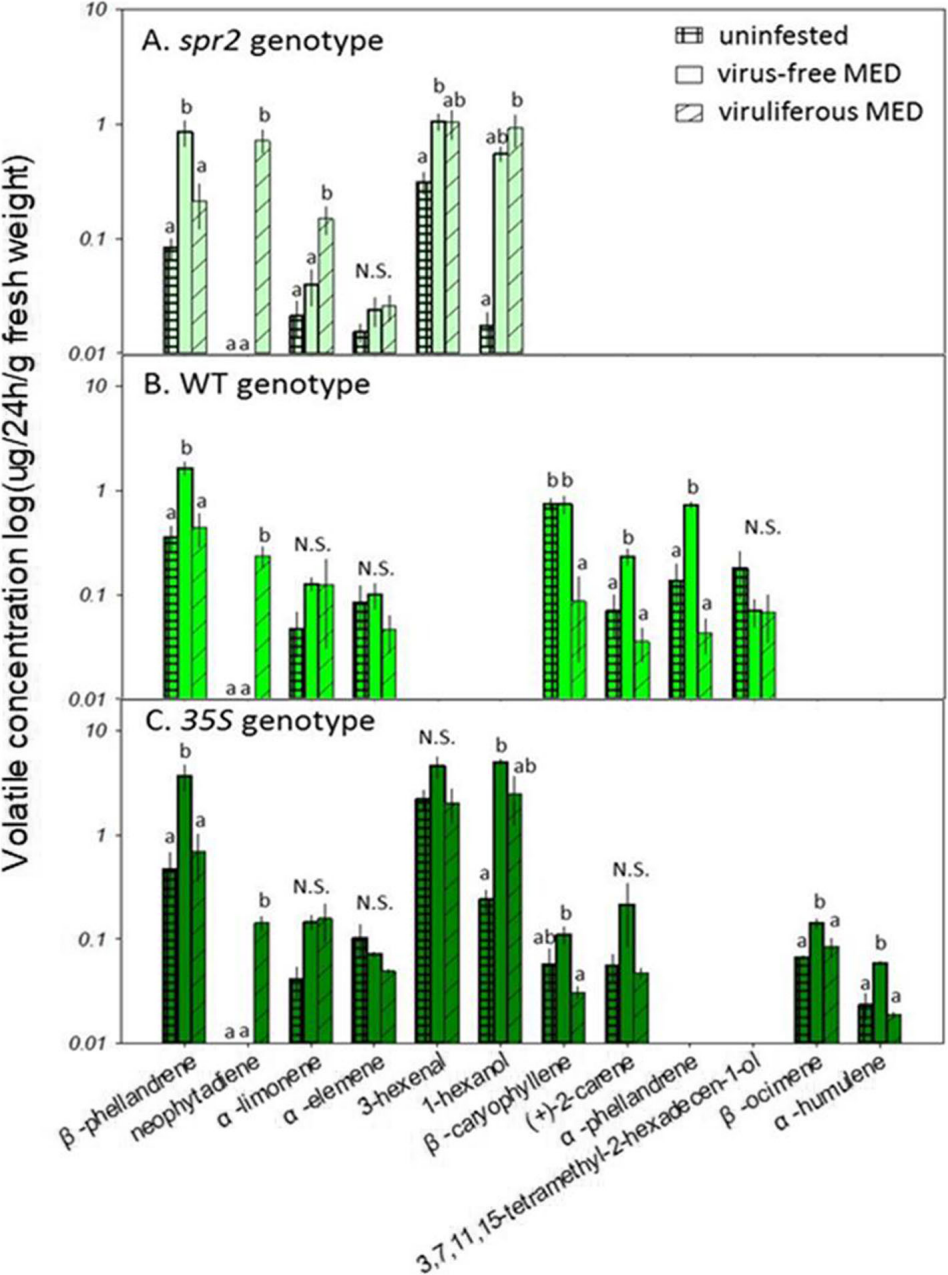
Plant volatile emissions as a function of genotype and prior MED infestation. **A ***spr2* plants. **B** WT plants. **C ***35S* plants. This protocol used 81 (three treatments × three genotypes × nine replicates) plants. Square grid bars: non-infested control plants; open bars: plants infested with virus-free MED; striped bars: plants infested with viruliferous MED. Within each group, bars with different letters are significantly different (*P* < 0.05)

We detected 12 volatile compounds, four of which (β-phellandrene, neophytadiene, α-limonene, and α-elemene) occurred in all three genotypes. Two compounds (3-hexanal, 1-hexanol) were found only in *spr2* and *35S* plants, two compounds (β-caryophyllene, (+)-2-carene) were found in WT and *35S* plants, and four compounds (α-phellanderene, 3,7,11,15-tetramethyl-2-hexadecen-1-ol, β-ocimene, α-humulene) were found in a single genotype (Fig. 4B, C). The low-JA *spr2* genotype had the fewest volatile compounds (six), while the WT and high-JA *35S* genotypes produced eight and ten compounds, respectively.

Neophytadiene was the only compound exclusively associated with whitefly infestation. It was found only in plants fed upon by viruliferous MED, in concentrations that were negatively correlated with plant JA levels (i.e., *spr2* > WT > *35S* plants, Fig. 4). Other than neophytadiene, there were no compounds consistently (i.e., in two or more plant genotypes) associated with viruliferous MED. Prior infestation by virus-free MED, however, increased β-phellandrene concentrations in all three plant genotypes (all *p* < 0.05), and 1-hexanol concentrations were observed in *spr2* and *35S* plants (Fig. 4A,C).

**Fig. 5:**
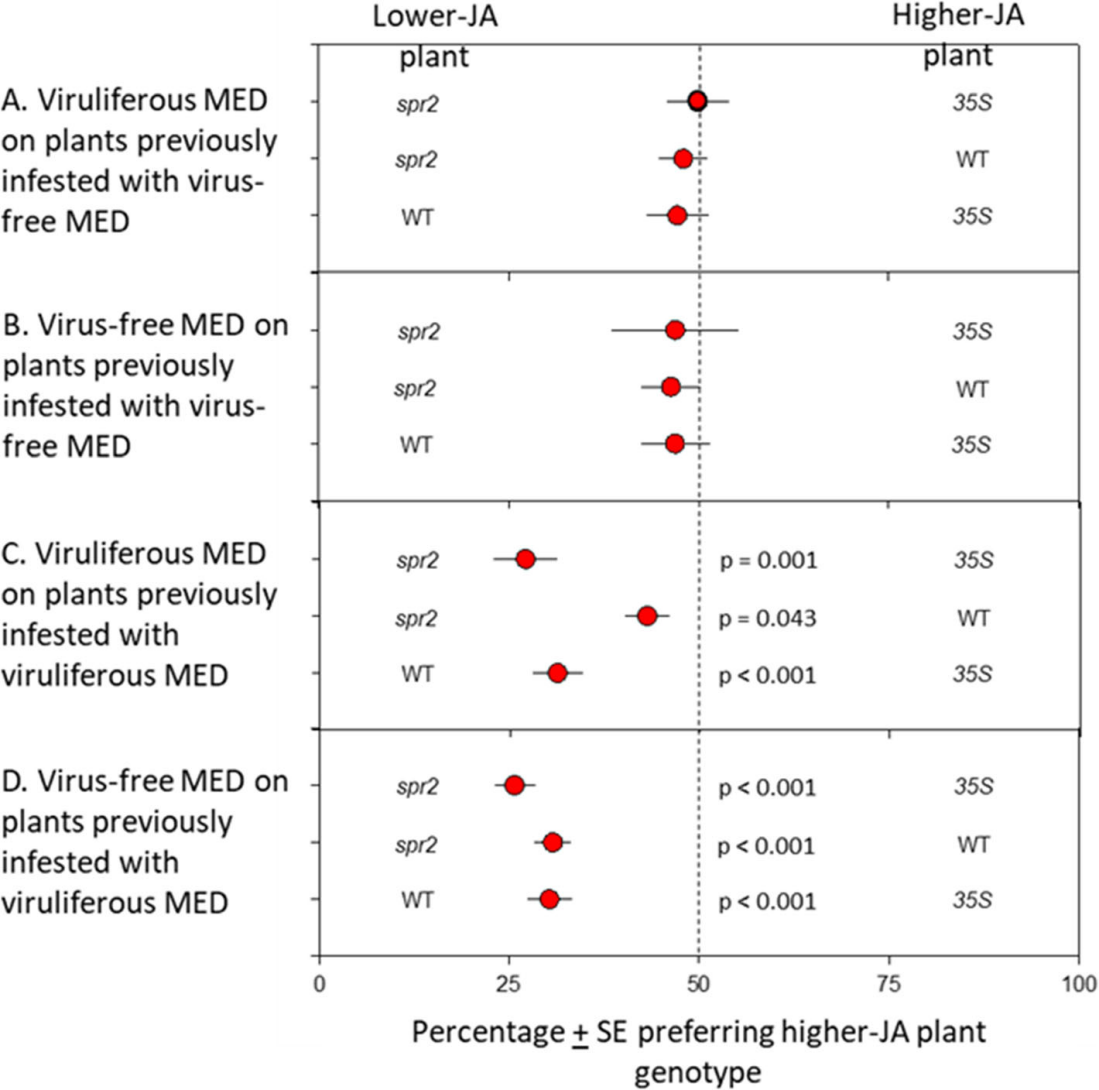
MED preference for different plant genotypes and prior infestation status. **A**. Virus-free MED choosing between plants of different genotypes that had both previously been fed upon by virus-free MED. **B**. Viruliferous MED choosing between plants of different genotypes that had both previously been fed upon by virus-free MED. **C.** Virus-free MED choosing between plants of different genotypes that had both previously been fed upon by viruliferous MED. **D.** Viruliferous MED choosing between plants of different genotypes that had both previously been fed upon by viruliferous MED

Neither virus-free nor viruliferous MED differentiated between plant genotypes that had previously been fed upon by virus-free MED (*p* > 0.05 for all; Fig. 5). In contrast, both virus-free and viruliferous MED strongly preferred lower-JA genotypes when choosing between two plant genotypes that had both been infested by viruliferous MED (*p* < 0.05 for all; Fig. 5C,D).

## Discussion

Our findings agree with work showing that JA pathways provide an effective defense against phloem-feeding herbivores. Both aphids and *B. tabaci* MEAM1*,* for instance, develop more quickly on JA-impaired versus JA-enhanced *Arabidopsis thaliana* genotypes (21, 22). We also found that both viruliferous MEAM1 and MED had decreased fecundity, longevity, and survival rates in JA-treated plants compared with control plants (Shi et al. 2017). However, here we found that JA-based defense did not affect TYLCV-carrying MED: while virus-free MED grew more slowly on higher-JA plants, viruliferous MED were unaffected by feeding on these plant genotypes (Fig. 1). This may result from differences between control plants treated with 1 mM JA spray in Shi et al. (2017) and high JA transgenic plants; the inhibitory effect of 1 mM JA may be much higher than that present in the high JA plants.

Feeding by virus-free MED increased expression of the JA-biosynthesis-related *LOX* and *OPR3* genes, although there was no corresponding increase in either JA levels or expression of the JA-induced genes *PI II* and *JAR1* (Fig. 2). These results contrast with previous research showing that virus-free MEAM1 suppresses herbivore-induced JA compared with undamaged controls (23, 24). The difference between our results and prior work may results from differences between tomato varieties used in the research and/or genotype difference between MEAM1 and MED. In contrast, viruliferous MED both decreased the expression of JA-related genes (Fig. 2A-D) and lowered JA levels (Fig. 2E, 3B) in WT and *35S* plants. Virus-induced suppression of this defense pathway has been documented in other systems: cabbage leaf curl virus suppressed JA expression in *A. thaliana* (25), for example, and co-infection of the begomovirus TYLCCNV and its beta-satellite suppressed JA-based defense against MEAM1 in tobacco (16). While both TYLCCNV and its beta-satellite are required to effectively suppress host defense, TYLCV is a true monopartite begomovirus that lacks a beta-satellite (26). Our results thus demonstrate that the monopartite begomovirus TYLCV can suppress even constitutively-expressed plant defenses and increase both vector and virus fitness. The impact of plant defenses on this suppression is shown by the fact that although viruliferous MED reduced JA levels in all three plant genotypes, TYLCV levels were still lower in *35S* plants than the other two genotypes (Fig. 3A, B). Lower viral titers in the JA-overexpressing line agree with work suggesting that JA can slow viral replication (5); activation of this defense pathway in *Phaseolus vulgaris*, for example, inhibited the potexvirus white clover mosaic virus (27).

When choosing between uninfested plants, both virus-free and viruliferous MED exhibited at least a marginal preference for the low-JA *spr2* over the high-JA *35S* genotype but did not differentiate between WT plants and either genotype. The preference for low-JA genotypes was stronger when whiteflies chose between plants that had both been fed upon by viruliferous MED (Fig. 5). The strongest preference of both virus-free and viruliferous MED was for *spr2* over *35S* (74% preference), but their low-JA preference was still significant when choosing between the JA-varying genotypes and WT plants. The enhanced preference for low-JA genotypes may be linked to the volatile compound neophytadiene, which was found only in plants previously infested with viruliferous MED. Neophytadiene concentrations (see Fig. 4) were highest in *spr2* plants (0.72 μg/24 h/g fresh weight), intermediate in WT (0.23 μg/24 h/g fresh weight), and lowest in *35S* plants (0.14 μg/24 h/g fresh weight), meaning that lower-JA plants were always higher in this compound. This agrees with other work showing that infestation by viruliferous MED induces neophytadiene production and that this volatile attracts MED (28); in addition, extracts from *Verbascum thapsus*, a plant high in neophytadiene, have also been found to attract whiteflies (29). In our experiment, whiteflies did not exhibit a preference for plants producing lower JA and higher neophytadiene between WT plants and either genotype, which suggests that other compounds may be involved. Four other terpenes, α-phellandrene, (+)-2-carene, β-caryophyllene, and α-humulene were induced by infestations of virus-free MED but reduced by viruliferous MED. All four compounds have been reported to exhibit repellent properties to whiteflies (30, 31), and their reduced expression in viruliferous MED plants likely reflects virally-induced reduction of plant defense.

By addressing how plant defense and its interaction with prior herbivory affects the host-vector-virus interaction, our results suggest several avenues for additional research. The ability of herbivorous insects to suppress host defenses and manipulate plant volatile emissions, for instance, often involves feeding-transmitted symbionts or compounds (32). Symbiotic microorganisms orally secreted by beetle larvae (*Leptinotarsa decemlineata*), for instance, suppress JA-derived defense and enhance larval growth (8); similarly, calcium-binding proteins in aphid saliva help suppress plant defense (33). Although similar processes may be at work in the MED-TYLCV-plant interaction, the molecular mechanisms such as the effectors underlying our results have not yet been elucidated. Finally, the interaction of neophytadiene and JA manipulation by viruliferous MED should be explored; the possibility that JA may affect neophytadiene induction may provide information critical for understanding this tripartite interaction and improving pest management.

## Conclusions

Our research highlights the complex interplay between plant defense, volatile emissions, and the host-vector-virus interaction. Though JA (or related metabolites) negatively affected both TYLCV and virus-free MED, it was ineffective against viruliferous MED. Feeding by viruliferous MED reduced JA levels and volatile terpene while inducing neophytadiene production. Future research into the relationship between MED, JA, and neophytadiene may illuminate new pathways for effective pest management.

## Methods

### Plants

Three tomato (*Solanum lycopersicum*) genotypes were used: *35S*: *prosystemin* plants that overexpress JA (*35S*), *spr2* plants that underexpress JA (*spr2*), and wild-type plants with normal JA levels (WT) (34, 35). The *35S*, *spr2*, and WT plants are all derived from the same parent genotype of tomato cv Castlemart. Tomato seedlings were grown in individual 1.5 L pots filled with potting mix (peat moss, vermiculite, organic fertilizer, and perlite in a 10:10:10:1 ratio by volume) in a glasshouse under natural light at 25–28 °C and 50–70% relative humidity.

TYLCV-infected plants were obtained by agro-inoculation of seedlings at the three true-leaf stage with a TYLCV genome (GenBank accession number: AM282874) originally isolated from tomato plants in Shanghai, China (36, 37). Infected plants developed characteristic leaf-curl symptoms; infection was confirmed by PCR with the primer set TYLCV-61 and -473 (12).

### Insects

Virus-free MED were originally collected in 2009 from *Euphorbia pulcherrima* growing near Beijing, China; they have since been maintained on virus-free *S. lycopersicum* within screen-mesh cages (0.6 **×** 0.6 **×** 0.6 m) in a greenhouse. Viruliferous MED were produced by confining 300 virus-free adults in cages with TYLCV**-**infected tomato plants. Viruliferous MED was confirmed by PCR with the primer set TYLCV-61 and -473 (12). Virus-free MED were obtained by confining 300 virus-free adults in cages with virus**-**free tomato plants. Both colonies were maintained for more than six generations in separate greenhouses under natural light at 25–28 °C and 50–70% RH. We confirmed that both MED colonies only contained MED by monitoring the *mitochondrial cytochrome oxidase I* (*mtCOI*) gene in 20 adults per generation (38).

#### Experiment I: impact of JA levels on virus-free and viruliferous MED

We assessed the preference and performance of virus-free and viruliferous MED on plant genotypes differing in JA expression: JA-deficient *spr2*, control WT, and overexpressing *35S*.

### MED preference

We conducted paired-choice experiments assessing whether host preference of virus-free and viruliferous MED was affected by plant genotype. Plants at the 6–7 true leaf stage from each of the three tomato genotypes were placed in whitefly-proof screen cages (80 × 40 × 60 cm), with two plants of different genotypes per cage. The two plants were placed in opposite corners of the cage, and at least 100 (mean 100.7 + 0.35 [SE] MED per cage across all replicates) virus-free or viruliferous MED that were the same age and had been starved for 24 h were released in the center. After one day, we covered each plant with transparent plastic wrap to prevent whiteflies from relocating and counted the number of MED per plant. For all three plant genotypes, each treatment (= two-plant combination) was replicated (= single cage) nine times.

### MED performance

We assessed nymphal survival (emerged adults/total eggs) and development time (days from egg to adult) by confining 20 adult MED (1:1 sex ratio) in a clip cage (30 mm in diameter; 20 mm in height) attached to the 3rd-6th true leaf from the top of a tomato seedling, one cage per plant, for 24 h (19, 39). Each plant was used only once, and each treatment was replicated 30 times (30 virus-free and 30 viruliferous MED replicates) per genotype. After 24 h, we removed the adults and used a stereomicroscope (Leica, M205C) to count egg production. On day 16, the first adult emerged; from that day onward, we collected emerging adults from each clip cage twice per day until all MED had matured. After all MED had matured, each whitefly-infested leaf was collected for quantification of TYLCV load using ELISA (40). Each treatment was replicated 30 times (30 virus-free and 30 viruliferous MED replicates) per genotype.

We assessed adult fecundity and longevity by transferring one newly-emerged virus-free or viruliferous female to a clip cage attached to the 3rd-6th true leaf from the top of a tomato seedling. Each plant was used only once, and each treatment was replicated 30 times (30 virus-free and 30 viruliferous MED replicates) per genotype. We took daily data on adult longevity and used a stereomicroscope to assess weekly egg production.

#### Experiment II: impact of plant genotype and TYLCV infection on expression of JA-related genes and JA levels

We quantified JA levels in plants (6–7 true leaf stage) from each of the three tomato genotypes. After we attached individual clip cages to six leaves per plant, all six cages per plant received one of the three following treatments: control (no MED in any of the cages), virus-free MED (50 virus-free adult MED per cage), or viruliferous MED (50 viruliferous adult MED per cage). The number of MED per plant in this experiment (50) was identified using a pilot experiment as the minimum number of MED necessary to affect JA levels in a 24-h period. Clip cages and whiteflies were removed after 24 h and the six leaves per plant collected. Plants treated with viruliferous insects were confirmed to be infected by PCR with the primer set TYLCV-61 and -473 (12). JA levels in each leaf were quantified using a gas chromatography-mass spectrometry system (Agilent Technologies, Santa Clara, CA) (41) and TYLCV loads were quantified using ELISA (40). This protocol used 162 (three infestation treatments × three genotypes × nine replicates × two determination category for JA level and TYLCV titer) plants.

We used the same protocol, but with three plants per treatment, to measure the expression of four JA-related genes: lipoxygenase (*LOX*), 12-oxophytodienoate reductase 3 (*OPR3*), proteinase inhibitor II (*PI II*), and JA-amino acid synthetase 1 (*JAR1*)*.* Both *LOX* and *OPR3* are involved in JA biosynthesis, with *LOX* controlling the initial oxygenation of α-linolenic acid, a fatty acid substrate (42), and *OPR3* catalyzing the reduction of the resulting 12-oxo-phytodienoic acid (OPDA; 43). Following biosynthesis, the presence of JA increases *JAR1* expression as well as production of proteinase inhibitors via up regulation of the *PI II* gene (43). *Actin* (*ACT*) and *ubiquitin 3* (*UBI*) (44) were used as reference genes (Table S1 in Supporting Information). Total RNA was extracted from 0.2 g of leaf tissue using an RNA extraction kit (Tiangen Biotech, Beijing, China), and 1.0 μg of RNA was used to synthesize the first-strand cDNA using the PrimeScript® RT reagent kit (Takara Bio, Tokyo, Japan) with gDNA Eraser (Perfect Real Time, TaKara, Shiga, Japan). The 25.0 μl reaction system contained 10.5 μl of ddH_2_O, 1.0 μl of cDNA, 12.5 μl of SYBR® Green PCR Master Mix (Tiangen Biotech, Beijing, China), and 0.5 μl of each primer. Relative RNA quantities were calculated using the comparative cycle threshold (2^-ΔΔCt^) method (45). Each treatment had 12 replicates (= 3 plants × 4 technical replicates) and used a minimum of four leaves per plant.

#### Experiment III: impact of plant genotype on TYLCV titer and JA levels following infestation with viruliferous MED

A single clip cage containing five viruliferous MED was placed on a plant at the three-true-leaf stage. Twelve healthy plants from each of the three tomato genotypes were exposed. The clip cages and viruliferous whiteflies were removed after two days of exposure, and the plants kept individually in insect-proof cages. The number of MED per plant in this experiment (5) reflects prior work assessing the number of MED necessary to reliably transfer TYLCV to plants. After 5 d, the first true leaf from each of the three plants per genotype was collected for quantification of virus titer using TAS-ELISA (40); this procedure was repeated at day 10, 20 and 30. Each plant was only sampled a single time, and was discarded after the leaf was removed. This protocol used 36 (three genotypes × three replicates × four time points) plants.

JA levels in plants were sampled one day before infestation and one day after infestation with viruliferous MED. Three leaves were sampled per plant for both pre- and post-infestation analyses, and JA concentrations were determined using a GC-MS as described in Experiment II. Each plant was used only once. This protocol used 18 (three genotypes × three replicates × two sampling dates) plants.

#### Experiment IV: impact of infestation by virus-free and viruliferous MED on volatile emissions from, and whitefly preference for, different plant genotypes

### Plant volatile emissions

Plants from each of the three tomato genotypes (*spr2*, WT, and *35S*) were exposed to one of three infestation treatments (clip cages with no whiteflies [=control], virus-free whiteflies, or viruliferous whiteflies) following the protocol detailed in experiment II. After two days, the clip cages and insects were removed from each plant and plant volatiles were collected using a slightly-modified version of the headspace collection system (6). Plant volatiles were collected for 6 h under continuous light, after which the whole plants were weighed (fresh weight, FW) to determine volatile quantity expressed per g FW. This protocol used 81 (three treatments × three genotypes × nine replicates) plants. TYLCV loads in leaves infested by virus-free and viruliferous whiteflies for each of the three plant genotypes was quantified using ELISA (40). This protocol used 81 (three treatments × three genotypes × nine replicates) plants.

We dissolved headspace samples in n-hexane, added 0.2 μg ml^− 1^ of n-dodecane to the solvent as an internal standard, then subjected a 1 μl sample to the HP-5MS column (60 m long, 0.25 mm diameter and 0.25 μm film thickness, Agilent Technologies, Santa Clara, CA) of gas chromatography–mass spectrometry. The true standards of the detected volatiles were also injected in different concentrations ranging from 0.2 to 50 μg ml^− 1^ hexane. Standard compounds were purchased from Beijing Huaerbo Technology Co., Ltd. The temperature profile was as follows: 50 °C for one min; 50 °C to 240 °C at 5 °C min^− 1^; 240 °C for two min; 240 °C to 300 °C at 30 °C min^− 1^; 300 °C for five min. The injection temperature was 270 °C, the source temperature was 200 °C, and the interface temperature was 280 °C. The column effluent was ionized by electron impact ionization (70 eV). Compounds were verified in the National Institute of Standards and Technology (NIST) database and mass spectra of the (co-) injected standards. Then compounds were quantified based on concentrations of true standards. All volatiles were analyzed separately.

### MED preference

We conducted a series of paired-choice experiments as per the protocols described in experiment I. Briefly, plants from each of the three genotypes were exposed to one of two infestation treatments (clip cages with either virus-free or viruliferous whiteflies) following the protocol detailed above. After two days, clip cages were removed and plants of different genotypes were placed in opposite corners of a screen cage. Each treatment (= two plants of different genotypes) was replicated nine times for 27 (= three two-genotype combinations × nine plants per combination) replicates in each paired-choice experiment. We conducted the following four paired-choice experiments:

A. Virus-free MED choosing between plants of different genotypes that had both previously been fed upon by virus-free MED.

B. Viruliferous MED choosing between plants of different genotypes that had both previously been fed upon by virus-free MED.

C. Virus-free MED choosing between plants of different genotypes that had both previously been fed upon by viruliferous MED.

D. Viruliferous MED choosing between plants of different genotypes that had both previously been fed upon by viruliferous MED.

#### Statistical analysis

For the paired-choice experiments, we used t-tests to assess whether virus-free or viruliferous whiteflies exhibited a preference for one plant genotype over another. For the performance experiment, we used two-way ANOVAs to assess the effect of MED infection status (virus-free, viruliferous), plant genotype (JA-deficient *spr2*, normal WT, overexpressing *35S*), and their interaction on whitefly life history parameters (development time, % survival to adulthood, longevity, and egg production) and whitefly preference. Data on survival from egg to adulthood was arcsine transformed prior to analysis to improve normality and homogeneity of variance.

Two-way ANOVAs were also used to compare the impact of MED infection status and plant genotype on endogenous JA levels and gene expression; gene expression data was square-root transformed prior to analysis where necessary.

We used repeated-measures ANOVA to assess whether plant genotypes differed in their virus titer following infestation with TYLCV, and whether plants differed in their pre- and post-infestation JA levels; for the time and genotype*time interactions, we report univariate unadjusted Epsilon F values. In cases where ANOVA revealed a significant main effect, Tukeys’ HSD (α = 0.05) was used to compare treatment means.

We analyzed the data on plant volatile emissions using one-way ANOVA to assess the effect of prior MED infestation (none, virus-free MED, viruliferous MED) on the concentrations of each volatile compound. In cases where there was a significant effect of MED infestation, Tukeys’ HSD (α = 0.05) was used to differentiate treatments. JMP 9.0.0 (SAS Institute, Durham NC) was used for all analyses.

## Data Availability

The datasets used and/or analyzed during the current study are available from the corresponding author on reasonable request.

## Abbreviations

ELISA: Enzyme linked immunosorbent assay
FW: Fresh weight
JA: Jasmonic acid
O. D: Optical delnsity
TYLCCNV: Tomato yellow leaf curl China virus
TYLCV: Tomato yellow leaf curl virus
